# DeltaDelta neural networks for lead optimization of small molecule potency†
†Electronic supplementary information (ESI) available. See DOI: 10.1039/c9sc04606b


**DOI:** 10.1039/c9sc04606b

**Published:** 2019-10-16

**Authors:** José Jiménez-Luna, Laura Pérez-Benito, Gerard Martínez-Rosell, Simone Sciabola, Rubben Torella, Gary Tresadern, Gianni De Fabritiis

**Affiliations:** a Computational Science Laboratory , Parc de Recerca Biomèdica de Barcelona , Universitat Pompeu Fabra , C Dr Aiguader 88 , Barcelona , 08003 , Spain . Email: gianni.defabritiis@upf.edu; b Laboratori de Medicina Computacional , Unitat de Bioestadística , Facultat de Medicina , Universitat Autònoma de Barcelona , Spain; c Janssen Research and Development , Turnhoutseweg 30 , 2340 Beerse , Belgium; d Biogen Chemistry and Molecular Therapeutics , 115 Broadway Street , Cambridge , MA 02142 , USA; e Pfizer I&I , 610 Main Street , Cambridge , MA 02139 , USA; f Acellera , Carrer del Dr Trueta, 183 , 08005 Barcelona , Spain; g Institució Catalana de Recerca i Estudis Avançats (ICREA) , Passeig Lluis Companys 23 , 08010 Barcelona , Spain

## Abstract

The capability to rank different potential drug molecules against a protein target for potency has always been a fundamental challenge in computational chemistry due to its importance in drug design. While several simulation-based methodologies exist, they are hard to use prospectively and thus predicting potency in lead optimization campaigns remains an open challenge. Here we present the first machine learning approach specifically tailored for ranking congeneric series based on deep 3D-convolutional neural networks. Furthermore we prove its effectiveness by blindly testing it on datasets provided by Janssen, Pfizer and Biogen totalling over 3246 ligands and 13 targets as well as several well-known openly available sets, representing one the largest evaluations ever performed. We also performed online learning simulations of lead optimization using the approach in a predictive manner obtaining significant advantage over experimental choice. We believe that the evaluation performed in this study is strong evidence of the usefulness of a modern deep learning model in lead optimization pipelines against more expensive simulation-based alternatives.

## Abstract 2

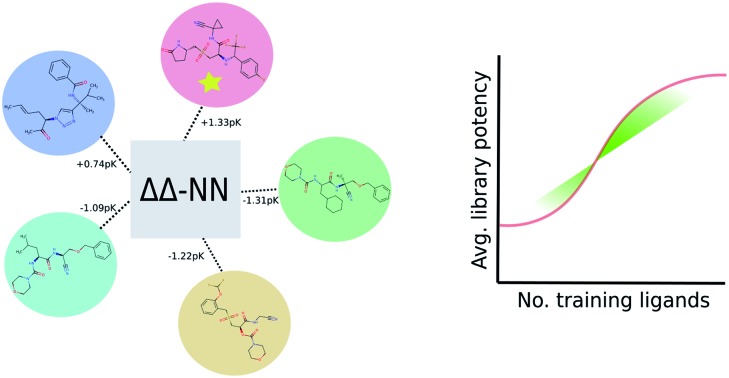
Machine learning approach tailored for ranking congeneric series based on 3D-convolutional neural networks tested it on over 3246 ligands and 13 targets.

## 1 Introduction

In the lead optimization phase of drug discovery, the chemical structure of a molecule is typically modified by a medicinal chemistry team with the intent of improving its potency, selectivity, and many other pharmacokinetic and toxicological parameters.^1^–^3^ These modifications result in congeneric series, a set of ligands with few atom changes between them, usually around a unique or small number of different scaffolds for which there are experimental structures of the complex with the target protein. Series range from few hundred to thousands of compounds and require considerable human, time and financial resources for synthesis and assays. It is therefore of great value to have *in silico* predictive tools to accelerate this process. Series typically feature very small potency differences, which in turn is a challenge for predictors, as having what could be considered a low error in other scenarios (*e.g.* below 1 kcal mol^–1^) is not a guarantee for successful ranking.

It is therefore common to focus on relative binding free energy (RBFE) simulation methods,^4^–^13^ where the difference in affinity between two ligands is computed using a thermodynamic cycle that alchemically perturbs only the small region associated with the changing atoms. RBFE methods have shown good results in several studies, with accuracy close to 1 kcal mol^–1^ and reasonable correlations. Despite this, these methods suffer from several issues, such as system preparation, treatment of waters, force-field selection, protein flexibility and computational cost, making their prospective application difficult in practice.^14^ On the other side, many empirical,^15^,^16^ knowledge-based^17^,^18^ and machine learning^19^–^24^ scoring functions have been designed for the task of predicting absolute binding affinities. They mostly tackle the problem in a regression setup, where the binding affinity is to be predicted using a set of protein–ligand descriptors, modelling the interaction among both. The fact that they model absolute affinities and are trained on very chemically diverse bodies of data, such as iterations of the PDBbind^25^ database, limits their applicability when predicting small structural differences between two ligands, such in the congeneric series case. While other machine learning approaches have been presented for this task,^26^–^28^ here we propose a modern 3D-convolutional-neural-network-based continuous learning approach for relative binding affinity prediction in congeneric series and show strong predictive power using multiple blind benchmarks as well as public datasets at negligible computational costs. This study serves as a very large evaluation of a modern machine-learning pipeline for lead optimization in a real-life drug discovery scenario, thanks to the joint collaboration with several pharmaceutical companies.

## 2 Materials and methods

### 2.1 Datasets studied

The BindingDB protein–ligand validation sets^29^ were used to pretrain our models. For testing, we also extracted well-known publicly-available literature test sets^30^ used for benchmarking RBFE calculations. Furthermore we include a recent freely-available BRD4 bromodomain dataset.^31^ In regards to internal pharmaceutical data, we tested on five different congeneric series from Janssen R&D. Three chemical series (sets 1, 2 and 3) were phosphodiesterase 2 (PDE2) inhibitors with bioactivity *versus* PDE2, PDE3, and PDE10 (ref. 32 and 33) (publication number WO2018083103A1), the fourth series were proto-oncogene tyrosine kinase (ROS1) inhibitors (publication number WO2015144799A1) and the final beta-secretase 1 (BACE1) inhibitors.^34^ We tested six congeneric series with Pfizer, three of which target a kinase, and the remaining an enzyme, a phosphodiesterase (PDE) and an activator of transcription. The sizes of these vary from 93 molecules up to 362, for a total of 955 tested compounds. Lastly, Biogen tested the proposed procedure on two different series, composed of 196 and 220 analogues targeting a tyrosine-protein kinase and a receptor-associated kinase, respectively. The size of the sets presented here allow, to the best of our knowledge the largest evaluation yet of a modern machine learning pipeline in lead optimization.

Out of the total 645 available congeneric series available in BindingDB, 495 with IC_50_ affinity values were extracted and processed for further evaluation, as it was the unit with most data available, containing a diverse set of targets. The majority of these sets encompass a single protein–ligand crystal structure, the rest of the ligands modelled against the reference using the Surflex docking software.^35^ We then assign each protein structure in the database to a family cluster using a 90% sequence similarity threshold, as per PDB conventions.^36^ For each series in the same protein cluster we use a maximum common substructure (MCS) protocol as available in rdkit^37^ to remove identical ligands. This procedure ensures that the same ligand is not repeated against similar targets, avoiding potential overfitting problems and overoptimistic evaluations.^38^ Affinity values were log-converted to avoid target scaling issues (pIC_50_ = –log_10_ IC_50_). Ligands that could not be read by rdkit were removed. Histograms of the number of ligands and their affinity range per series are provided in ESI Fig. 7,† with the average available number of ligands per series being 8.84. In the Schrödinger and BRD4 sets, since only Δ*G* (per kcal mol^–1^) information was available, we converted affinity values to the pIC_50_ range assuming non-competitive binding. Descriptive information on these series is provided in ESI Table 1.† Compounds provided by Janssen were docked using a common scaffold structure *via* the Glide software. These congeneric series range from 48 up to a 900 different compounds with varying affinity ranges (ESI Table 2†).

### 2.2 Descriptor calculation

We have recently reported a machine learning approach that can learn based on 3D features of the binding site interactions.^20^ A similar encoding was used here that represents the protein–ligand binding by voxelizing both using a 24 Å pocket centered box, with a density of 1 Å^3^ per voxel. The contribution of each atom to each voxel is inversely proportional to their Euclidean distance *r* and the van der Waals radius *r*_vdw_ of the first. We use several *channels* for both protein and ligand, in the sense that the atomic contribution to each voxel depends on their type. The contribution of each atom to each voxel is assigned according to a pair correlation function defined by:1
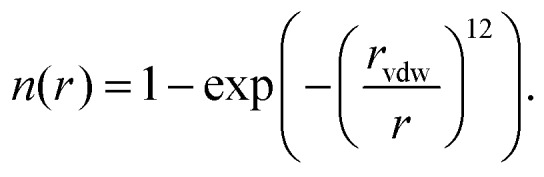



We define several *channels* for both protein and ligand, in the sense that the atomic contribution to each voxel depends on their type. For the protein we define eight pharmacophoric-like descriptors, as detailed in ESI Table 3.† For the ligands we use a simpler representation based on atom types contained in the set {C,N,O,F,P,S,Cl,Br,I,H}, for a total of 18 stacked channels. We note that there is no particular reasoning behind this choice of descriptors other than they showed promising practical performance in previous studies.

### 2.3 Neural network architecture

Regular feed-forward neural networks do not scale well when the input is high dimensional (as in images, or in this case atomic interactions). CNNs are specifically designed for handling lattices, where local spatial information needs to be preserved. While a feed-forward network would ignore such interactions, a convolutional one arranges its neurons spatially, and only connects locally to the output of the previous layer. The latter type of architectures have become the de-facto workhorse in computer vision problems,^39^–^41^ providing state-of-the-art performance. Due to this success, many applications in bioinformatics and computational chemistry followed.^42^–^51^

The neural network we propose has a novel zero-symmetric architecture whose main building blocks are 3D-convolution operations. In this work we focus on predicting relative affinities for close analogues in lead optimization, therefore, our approach is to build a network whose input is a pair of ligand binding voxelized representations belonging to the same series. A two-input convolutional neural network was designed, with fixed weights on both legs. The inputs are forwarded through several convolution and pooling operations and then flattened into a 192-dimensional latent representation. The symmetry property of relative binding affinity requires that inverting the order of the input ligands should change the sign of the predicted value, and we embed such symmetry in the network by computing the difference between latent representations. A final linear layer with no bias is then applied to the result of this difference, ensuring zero-symmetry by design and producing the desired predicted difference in affinity. In contrast, calculating relative affinities from an absolute predictor inevitably leads to the concatenation of errors from two separate predictions.

**Fig. 1:**
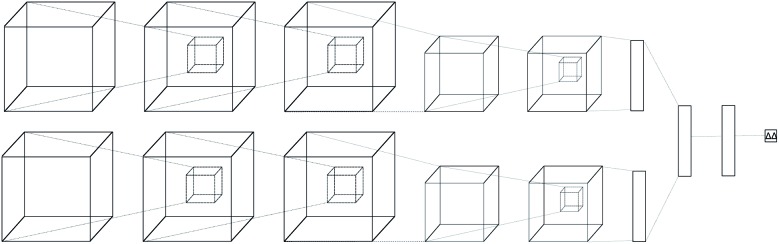
Architecture of the proposed model. A two-legged neural network with tied weights was constructed, and a pair of protein–ligand voxelization is feed-forwarded through it to later perform a latent space difference.

A schema of our architectural choice is provided in Fig. 1 is provided in the ESI.† It features two convolution operations with a kernel size of 3 in each leg, followed by a max-pooling operation, and finally another convolution operation with the same kernel size for both before flattening and performing the latent difference between analogues. The ReLU activation function was used for all layers in the network except for the last, which does not feature one. We include a dropout layer in the end to control for overfitting. Xavier initialization was used for the weights. Training is performed using the Adam stochastic gradient descent optimizer^52^ with standard hyperparameters (*β*_1_ = 0.9, *β*_2_ = 0.999, *ε* = 10^–4^) using a batch size of 32 samples for 50 epochs. Data augmentation is performed during training by randomly rotating input coordinates to mitigate the lack of rotational equivariance in CNNs. Furthermore, given a set of relative binding predictions, its absolute counterparts can always be retrieved given a single experimentally determined absolute reference, such as the one provided by a lead. If more than one is available, absolute affinities can be computed towards each, in practice providing a predictive absolute affinity distribution, whose average can then be interpreted as a maximum *a posteriori* (MAP) estimate of the absolute affinity and its standard deviation as a measure of its uncertainty, given the current model state.

### 2.4 Continuous learning

In the proposed continuous-learning approach, we explicitly use the fact that congeneric series are sequentially generated in a lead optimization campaign, and follow an incremental training and testing procedure. For each congeneric series at a given time the affinity of previously tested ligands is known experimentally: differences for these are taken as training data, while for test data we predict differences between unknown and known ones. While this approach is less ambitious than having a predictor for relative affinity with no experimentally tested data (such as a physical-based model), its applicability is general, since it is the common scenario that medicinal chemists face in lead optimization campaigns. The training for the BindingDB sets starts with a reference structure in each series, for which we take the crystal structure ligand if available or the structure with the lowest average maximum common substructure (MCS) distance to the rest. Ligands from the rest of the series are then sequentially added in a random order. It is well known that either a random^53^ or scaffold-based training test split produce overoptimistic results when testing machine-learning algorithms on activity benchmarks. Since the industrial datasets in our study include a compound creation time-stamp, we also evaluate a more realistic temporal split,^54^ where at each training step we consider the first *n* tested ligands and the differences of the posterior ones against the first are taken. The performance of the machine learned models is reported as the root mean squared error (RMSE) and either Pearson's correlation coefficient *R* or Spearman's *ρ* between experimental and predicted affinity differences. We note that in all blind tests a single model was provided, and no explicit attempt to optimize hyperparameters in each set was made.

## 3 Results

We first present results concerning our validation on the 495 protein-series datasets from the BindingDB, where the proposed model achieves an average correlation coefficient above 0.4 and an RMSE below 1.25 (pIC_50_ units) even when only one binding-energy difference is taken per congeneric series (ESI Fig. 1†). This suggests that the method works reasonably well in the very low-data scenario, such as the beginning of a lead optimization campaign. A noticeable performance boost is seen as more differences are included in training, with a correlation coefficient above 0.62 and an RMSE below 1.05 when another four different ligands from the same congeneric series are known in advance, with performance plateauing beyond five additional training ligands. A comparison against an absolute affinity model is also provided (*i.e.* one of the legs of the architecture), where as expected it can be appreciated that it performs considerably worse than its relative counterpart.

Now we present results on the Wang *et al.*^30^ and BRD4 inhibitor datasets.^31^ In this and the rest of cases, we pretrained a model with all difference pairs available in the BindingDB database, which provides a prior for further fine-tuning. We then mixed new available data as training in each sequential iteration of each set with the rest of the BindingDB database for only 3 epochs, significantly reducing computational overhead. A FEP baseline provided by Wang *et al.*^30^ is used for comparison. The model efficiently interpolates differences for unseen ligands, achieving considerably high correlation coefficients and low errors in all series with as few as 3–4 additional ligands and associated activity pairs, surpassing in many cases the much more expensive FEP baseline (ESI Fig. 2†). For instance, for the MCL1 target, after testing 3 ligands, the correlation coefficient is above 0.8, surpassing the FEP baseline, and the RMSE is below 1.2 (pIC_50_ units).

**Fig. 2:**
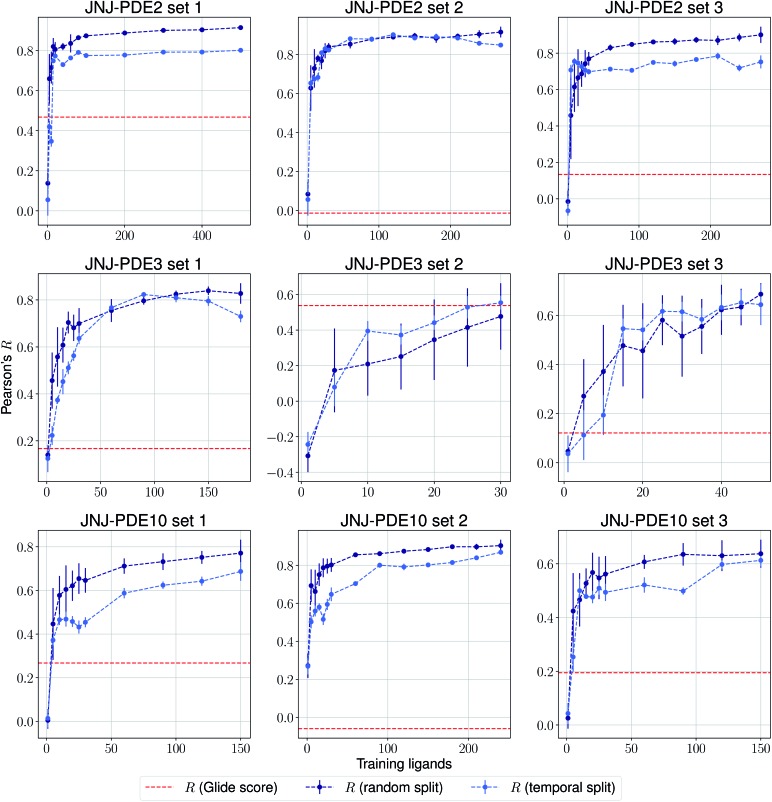
Average Pearson's correlation coefficient *R* (±1 standard deviation) based on 25 independent runs on different sets for the Janssen PDE2, PDE3 and PDE10 targets.

**Fig. 3:**
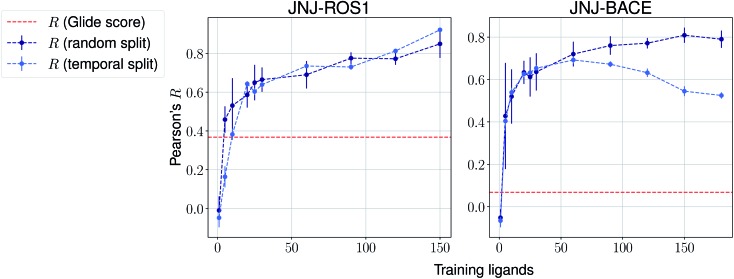
Average Pearson's correlation coefficient *R* (±1 standard deviation) based on several independent runs on two sets for the Janssen ROS1 and BACE targets.

The same evaluation procedure was taken for the compounds available in the Janssen PDE sets (Fig. 2 and ESI Fig. 3†) for both a random and a temporal split, where a baseline against Glide score^55^ is also added. Excellent performance was seen on a random split given enough training data, and as expected, although the temporal split performance is lower, it is still sufficiently high to be used in a real-life prospective lead optimization scenario. For instance, for the first PDE2 activity set after 20 ligands sorted by time, the Pearson's correlation coefficient *R* and RMSE were 0.77 and 1.35 (in pIC_50_ units) respectively. Results for the ROS1 and BACE sets, show a similar trend and insights (Fig. 3 and ESI Fig. 4†). Interestingly, performance slightly decreases for the BACE target late in the evaluation, suggesting that the network may have found an unexplored activity cliff or that the last tested ligands are harder to predict than others in the series. Furthermore, we also provide a type of split where only differences among the most chemically close ligands are predicted, based on ECFP4 fingerprint similarity, as available in rdkit. That is, in each training step we predict from the remaining untested pool of ligands those that are closest to the ones in our training set, with the intention of resembling a real-life lead optimization RBFE scenario, typically applied to close analogues. Split-based results on fingerprint similarity for the first PDE2 set (ESI Fig. 5†), show that after 20 ligands sorted by chemical similarity the *R* and RMSE were 0.83 and 1.12 (in pIC_50_ units). These suggest better performance in this scenario than the proposed temporal split, and closer to the random one.

**Fig. 4:**
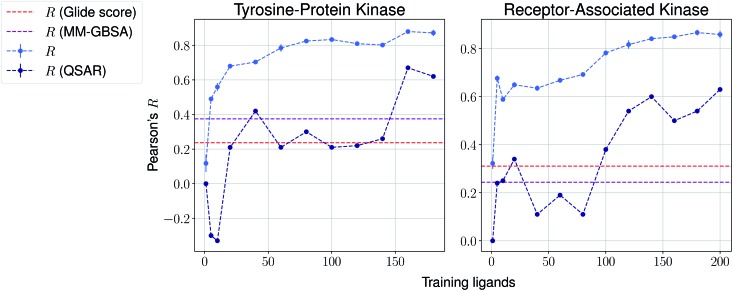
Results over 5 runs on Biogen's tyrosine-protein kinase and receptor-associated kinase using a temporal split, and MM-GBSA and QSAR random forest pipelines as baselines.

We then present the results provided by Pfizer using a temporal split in Table 1, where specific target names cannot be disclosed. We compare such results with several baselines such as molecular weight, clog *P*, a MM-GBSA pipeline^56^,^57^ and deep-learning absolute affinity predictor *K*_DEEP_,^20^ trained on the v.2016 iteration of the PDBbind database. The model proposed here performs considerably better than the rest when given only 10% of the training data, again highlighting the importance of incrementally training these on the congeneric series of interest. An exception, however, is found in the Kinase #3 series, for which no significant improvement is observed when providing extra training data. This particular last case appears to be particularly hard to predict, as all tested methods perform poorly. We provide results using a temporal split for the last two congeneric series provided by Biogen, for which we also compare against several baselines: (a) Glide score, (b) an MM-GBSA pipeline, and (c) a standard QSAR approach using MACCS, ECFP4 and rdkit descriptors with a random forest model (Fig. 4 and ESI Fig. 6†). Our model reveals similar conclusions, significantly outperforming all baselines. Curiously, it can also be seen that the proposed method does not perform significantly worse than the aforementioned baselines in the second target when no training data is used. When some is used, such as only 5 analogues, our proposed machine-learning model significantly outperforms all baselines.

**Table 1:** Spearman's *ρ* performance results between experimental and predicted absolute affinities provided by Pfizer I&I, where other empirical, simulation, and machine-learning based affinity prediction methods are compared on several congeneric series. Performance is poor for most tested model except for the sequential approach proposed here, with Pearson correlations averaging over 0.5 with as few as 10% used analogues from the congeneric series at hand

Target	# ligands	Mol. weight (*ρ*)	clog *P*^*a*^ (*ρ*)	MM-GBSA (*ρ*)	*K*_DEEP_ (*ρ*)	This work (10% training, *ρ*)	This work (20% training, *ρ*)	This work (30% training, *ρ*)
Kinase #1	362	0.19	0.06	0.56	0.42	0.49	0.64	0.73
Kinase #2	106	0.1	0.28	0.25	0.25	0.25	0.41	0.51
Kinase #3	95	0	0.04	0.25	–0.27	0.3	0.3	0.31
Enzyme	93	0.43	0.24	0.01	0.49	0.43	0.26	0.59
Phosphodiesterase	100	0.37	0.36	0.67	0	0.49	0.64	0.73
Activator of transcriptions	199	0.13	0.08	0.66	0.29	0.72	0.84	0.94
Weighted avg.		0.19	0.14	0.47	0.25	**0.49**	**0.59**	**0.69**
Simple avg.		0.2	0.18	0.4	0.18	**0.45**	**0.52**	**0.64**

^*a*^Calculated log *P* as available in rdkit.

All the tests regarding internal pharmaceutical data were carried out blindly by providing fully-containerized software to our collaborators, who executed the application and reported corresponding results. Furthermore only one pretrained model was provided without any opportunity to overfit to each specific test set.

One aim of our study was to test whether machine-learning driven relative affinity predictions could efficiently identify key high potency compounds in a close to real-life lead optimization scenario, by retrospectively comparing them to the experimental order of synthesis. With some of the large industrial datasets it was possible to test this and we used the most active compound as a surrogate interesting lead molecule. The model is trained on the first experimentally tested compounds, and then is incrementally trained by choosing from the remaining ones based on a upper confidence bound (UCB)-like criterion,^58^ defined as:

2 UCB = *μ*(*x*) + *βσ*(*x*),

where *μ* and *σ* are the average and standard deviation predicted absolute affinities provided by the model for ligand *x* and *β* is a user-chosen factor controlling the balance between exploitation and exploration, that we fix in our study to *β* = 1.64.

**Fig. 5:**
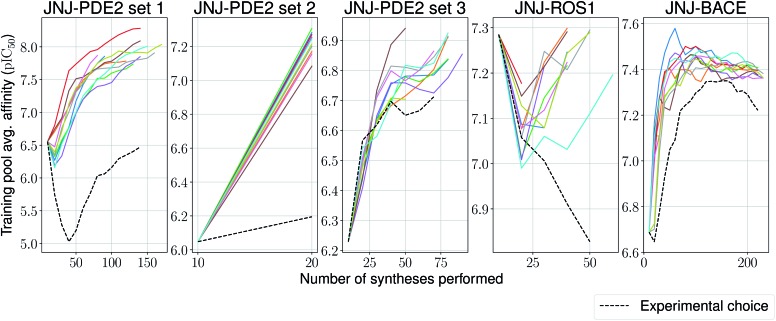
Average model-picked training set affinity per number of compounds synthesized for the Janssen PDE2, ROS1 and BACE sets, as well as a baseline based on the actual experimental choice order of compounds.

We stop the procedure once the model retrieves the analogue with the highest associated affinity, and compare this with its original synthesis experimental order in its corresponding series. We present results for this simulation-based benchmark in Table 2. In 4 out of 5 sets our proposed model is able to reach the compound with the highest affinity faster than its experimental order or by random selection. Surprisingly, in all ten independent runs of the second set for the PDE2 target, the compound with the highest affinity was found after only a single synthesis epoch. Furthermore, one would expect the average affinity in the training set to increase at each synthesis epoch (as the model is tasked to pick compounds with increasingly higher UCB). This is the case for 4 out of 5 sets again (Fig. 5), with the exception of the ROS1 target, which shows a non-monotonic trend, albeit its model reaches the compound with highest affinity before its experimental order. In all tested cases, the average training pool affinity for the ligands selected by the model is higher than experimental choice. Overall results are very promising and suggest that the proposed method could be applied successfully in a prospective scenario. Particularly, in the first PDE2 set, we were able to reach potent compounds synthesizing up to six times less molecules than the baseline method used by the medicinal chemistry team.

**Table 2:** Simulation-based benchmark results over 10 independent runs for the different datasets. We show the amount of molecules the model is allowed to pick at each synthesis epoch, the experimental order of the compound with the highest affinity in the series, the average synthesis epoch our model found said molecule, the total necessary sampled ligands the proposed model has chosen before the target compound, and the sampling advantages over the experimental and random orders

Target	Set	# ligands	Chosen per synthesis epoch	Experimental order	Found at synthesis epoch	Total sampled ligands	Advantage over experimental choice	Advantage over random choice
PDE2	1	900	10	766	12.2	132	**634**	**318**
PDE2	2	303	10	61	1	20	**41**	**131.5**
PDE2	3	278	10	253	5.9	69	**184**	**70**
ROS1	—	165	10	73	3.1	41	**32**	**41.5**
BACE	—	229	10	190	20.8	218	–28	–103.5

## 4 Discussion

In this work we have designed and tested a deep-learning based model for the task of predicting relative binding affinity predictions in congeneric series. This work provides evidence that the method is able to efficiently rank compounds as shown by an evaluation on both publicly available and industrial data and can be of use by computational and medicinal chemists in early drug-discovery projects by providing informed choices of future compounds to synthesize, as suggested by our simulation-based benchmark. The accuracy of the method heavily depends on the amount of available data but can be trained and applied in minutes on a single GPU, offering a substantial improvement in performance compared with physics-based RBFE calculations which can take days for a small number of analogues. While the results presented here are encouraging, it is important to note that they remain retrospective: a proper prospective validation of the model, which would entail chemists synthesizing compounds according to the decisions taken by the trained model, remains a topic of future study. In the long term, however, we expect that improving molecular simulations accuracy^59^,^60^ by the integration of physics and machine learning approaches would produce a more convenient approach for engineering drug discovery. In the meantime, methods such as the one proposed here provide accurate performance at a fraction of the computational cost of other approaches.

## Code & data availability

All the models here were developed using the PyTorch package for tensor computation and neural network training.^61^ BindingDB, Wang *et al.* and Mobley *et al.* set results are available upon reasonable request. Python code for generating the proposed featurization is available within the open-source HTMD software.^62^ The code of the network architecture in a PyTorch implementation is provided in the ESI.† An implementation of this application is available through the ; http://PlayMolecule.org repository of applications, where users can freely submit their protein in PDB format and two sets of the same congeneric series, for training and validation respectively in SDF format. Depending on the size of these last two, training and prediction time may vary, as the order of data for training increases by 
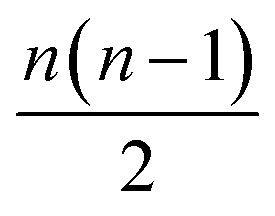
, and for testing *nm* factors, where *n* and *m* are the number of training and testing instances respectively. At the moment, predictions are limited to a default total of a 1000 molecules per congeneric series, with runtimes averaging and hour on a modern GeForce 1080Ti GPU. Larger experiments can be arranged for users willing to run more computationally demanding experiments.

## Conflicts of interest

G. D. F is a founder and current CEO of Acellera Ltd. J. J. receives funding from Acellera Ltd. and G. M. R. is an employee.

## Supplementary Material

Supplementary information
