# Supplementary material for: DeltaDelta neural networks for lead optimization of small molecule potency
Source: Chem Sci. 2019 Oct 16;10(47):10911–8. doi: 10.1039/c9sc04606b (PMC7066671; doi:10.1039/c9sc04606b)
Supplement: Supplementary file 1 [file SC-010-C9SC04606B-s001.pdf]

Electronic Supplementary Material (ESI) for Chemical Science.
This journal is © The Royal Society of Chemistry 2019

DeltaDelta Neural Networks for Lead Optimization of Small Molecule Potency
Supplementary Information

José Jiménez-Luna[1], Laura Pérez-Benito[2, 3], Gerard Martínez-Rosell[4], Simone Sciabola[5], Rubben Torella[6], Gary Tresadern[3], and Gianni De Fabritiis[1, 4, 7, *]

[1]Computational Science Laboratory, Universitat Pompeu Fabra, Parc de Recerca Biomèdica de Barcelona, C Dr. Aiguader 88. Barcelona, 08003, Spain.
[2]Laboratori de Medicina Computacional, Unitat de Bioestadística, Facultat de Medicina, Universitat Autònoma de Barcelona, Spain.
[3]Janssen Research and Development, Turnhoutseweg 30, 2340 Beerse, Belgium.
[4]Acellera, Carrer del Dr. Trueta, 183, 08005 Barcelona, Spain.
[5]Biogen Chemistry and Molecular Therapeutics, 115 Broadway Street, Cambridge, MA 02142, USA.
[6]Pfizer I&I, 610 Main Street, Cambridge, MA 02139, USA.
[7]Institució Catalana de Recerca i Estudis Avançats (ICREA), Passeig Lluis Companys 23, 08010 Barcelona, Spain.
[*]E-mail: gianni.defabritiis@upf.edu

Listings

1 Two-legged three-dimensional convolutional neural network architecture used in this study. Weights are fixed in both legs, and a difference in latent space (representing difference in binding) is then performed. 3

List of Figures

1 Average Pearson's correlation coefficient R and RMSE (±1 standard deviation) based on 25 independent runs on the BindingDB protein-ligand validation sets, with varying number of ligands in training and test and comparison against an identically-trained absolute binding affinity predictor. 5
2 Average Pearson's correlation coefficient R and RMSE (±1 standard deviation) based on 25 independent runs on the Schrödinger dataset [1] and BRD4 inhibitor series [2] with reference reported FEP performance. For the BRD4 series, an average FEP performance between two studies was taken for reference [3, 4]. 6
3 Average Pearson's correlation coefficient R and RMSE (±1 standard deviation) based on 25 independent runs on the Janssen PDE sets, using both a random and a temporal split and a Glide score baseline. 7
4 Average Pearson's correlation coefficient R and RMSE (±1 standard deviation) based on 25 independent runs on the Janssen ROS1 and BACE sets, using both a random and a temporal split and a Glide score baseline. 8
5 Pearson's correlation coefficient R and RMSE (±1 standard deviation) based on 25 independent runs on the Janssen PDE2 first set using a chemical similarity based split. 8

6 Average Pearson's correlation coefficient R and RMSE (±1 standard deviation) based on 5 independent runs on the Biogen Tyrosine-Protein Kinase and Receptor-Associated Kinase sets, using temporal split and with MM-GBSA, Glide score, and a random forest QSAR model (MACCS + ECFP4 + rdkit descriptors) as baselines. 9
7 Histograms of available ligands and range per congeneric series in the BindingDB database. 10

List of Tables

1 Descriptive information regarding the complexes taken from Wang *et al.* and Mobley *et al.* 4
2 Descriptive information regarding the series from Janssen R&D. 4
3 Rules defined for the 3-dimensional descriptors described in this work. 4

```python
import torch
import torch.nn as nn
import torch.nn.functional as F
from torch.autograd import Variable


def weights_init(m):
    """
    Xavier weight init.
    """
    if isinstance(m, nn.Conv3d) or isinstance(m, nn.Linear):
        torch.nn.init.xavier_normal(m.weight.data)
        if m.bias is not None:
            m.bias.data.fill_(0)


class DeltaDeltaG(nn.Module):
    """
    Delta-delta G simple prototype.
    """
    def __init__(self, input_channels=16):
        super(DeltaDeltaG, self).__init__()
        self.conv1 = nn.Conv3d(input_channels, 32, kernel_size=3)
        self.conv2 = nn.Conv3d(32, 32, kernel_size=3)
        self.max1 = nn.MaxPool3d(3)

        self.conv3 = nn.Conv3d(32, 3, kernel_size=3)
        self.lin1 = nn.Linear(192, 1, bias=False)
        self.dp1 = nn.Dropout(p=.5)
        self.apply(weights_init)

    def forward_once(self, x):
        x = F.relu(self.conv1(x))
        x = F.relu(self.conv2(x))
        x = self.max1(x)

        x = F.relu(self.conv3(x))
        x = x.view(x.shape[0], -1)
        return x

    def forward(self, x0, x1):
        d1 = self.forward_once(x0)
        d2 = self.forward_once(x1)
        diff = self.dp1(d1 - d2)
        return self.lin1(diff)
```

Listing 1: Two-legged three-dimensional convolutional neural network architecture used in this study. Weights are fixed in both legs, and a difference in latent space (representing difference in binding) is then performed.

Supplementary Table 1: Descriptive information regarding the complexes taken from Wang *et al.* and Mobley *et al.*

**Target**	**PDB Id.**	**# ligands**	**Affinity range (kcal / mol)**
BRD4	(a)	8	4.46 [a]
BACE	4DJW	36	3.5
CDK2	1H1R	16	4.2
JNK1	2GMX	21	3.4
MCL1	4HW3	42	4.2
P38	3FLY	34	3.8
PTP1B	2QBS	23	5.1
Thrombin	2ZFF	11	1.7
TYK2	4GIH	16	4.3

[a]Complexes taken from github.com/MobleyLab/benchmarksets, with experimental and FEP validation provided in several publications [3, 4]

Supplementary Table 2: Descriptive information regarding the series from Janssen R&D.

**Target**	**Set no.**	**# ligands**	**Affinity range (pIC_{50})**
PDE2	1	900	5.78
PDE2	2	303	4.66
PDE2	3	278	3.86
PDE3	1	218	3.57
PDE3	2	48	1.85
PDE3	3	65	2.02
PDE10	1	166	3.22
PDE10	2	270	3.18
PDE10	3	216	2.39
ROS1	-	165	3.39
BACE	-	229	3.89

Supplementary Table 3: Rules defined for the 3-dimensional descriptors described in this work.

**Property**	**Rule**
Hydrophobic	Aliphatic or aromatic C
Aromatic	Aromatic C
Hydrogen bond acceptor	Acceptor 1 H-bond or S Spherical N<br>Acceptor 2 H-bonds or S Spherical O<br>Acceptor 2 H-bonds S
Hydrogen bond donor	Donor 1 H-bond or Donor S Spherical H with either O or N partner
Positive ionizable	Gasteiger positive charge
Negative ionizable	Gasteiger negative charge
Metallic	Mg, Zn, Mn, Ca or Fe
Excluded volume	All atom types

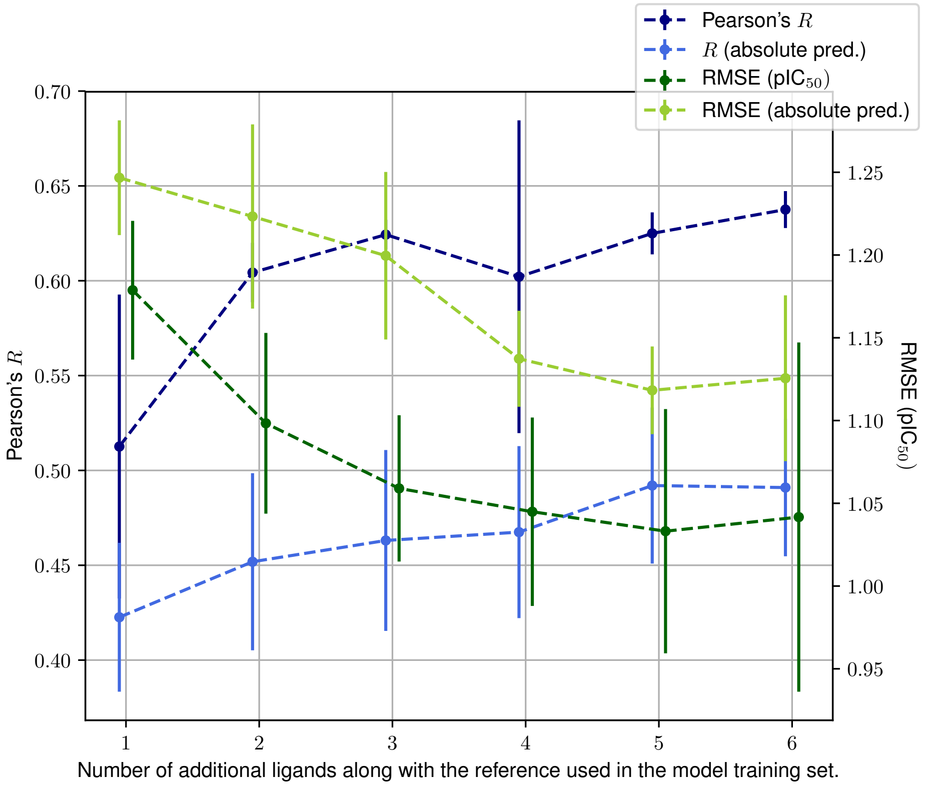

Supplementary Figure 1: Average Pearson's correlation coefficient R and RMSE (± 1 standard deviation) based on 25 independent runs on the BindingDB protein-ligand validation sets, with varying number of ligands in training and test and comparison against an identically-trained absolute binding affinity predictor.

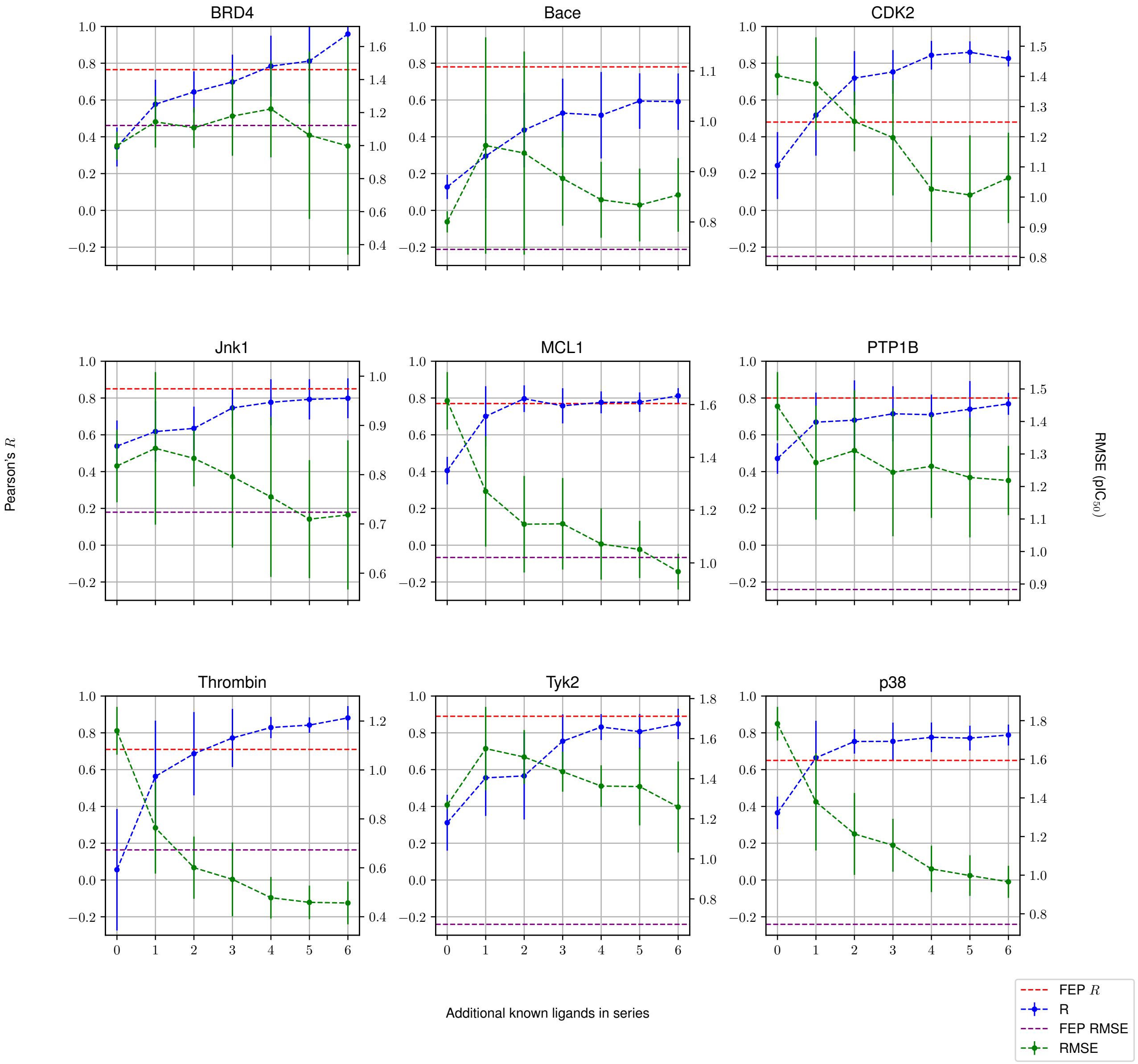

Supplementary Figure 2: Average Pearson's correlation coefficient R and RMSE (± 1 standard deviation) based on 25 independent runs on the Schrödinger dataset [1] and BRD4 inhibitor series [2] with reference reported FEP performance. For the BRD4 series, an average FEP performance between two studies was taken for reference [3, 4].

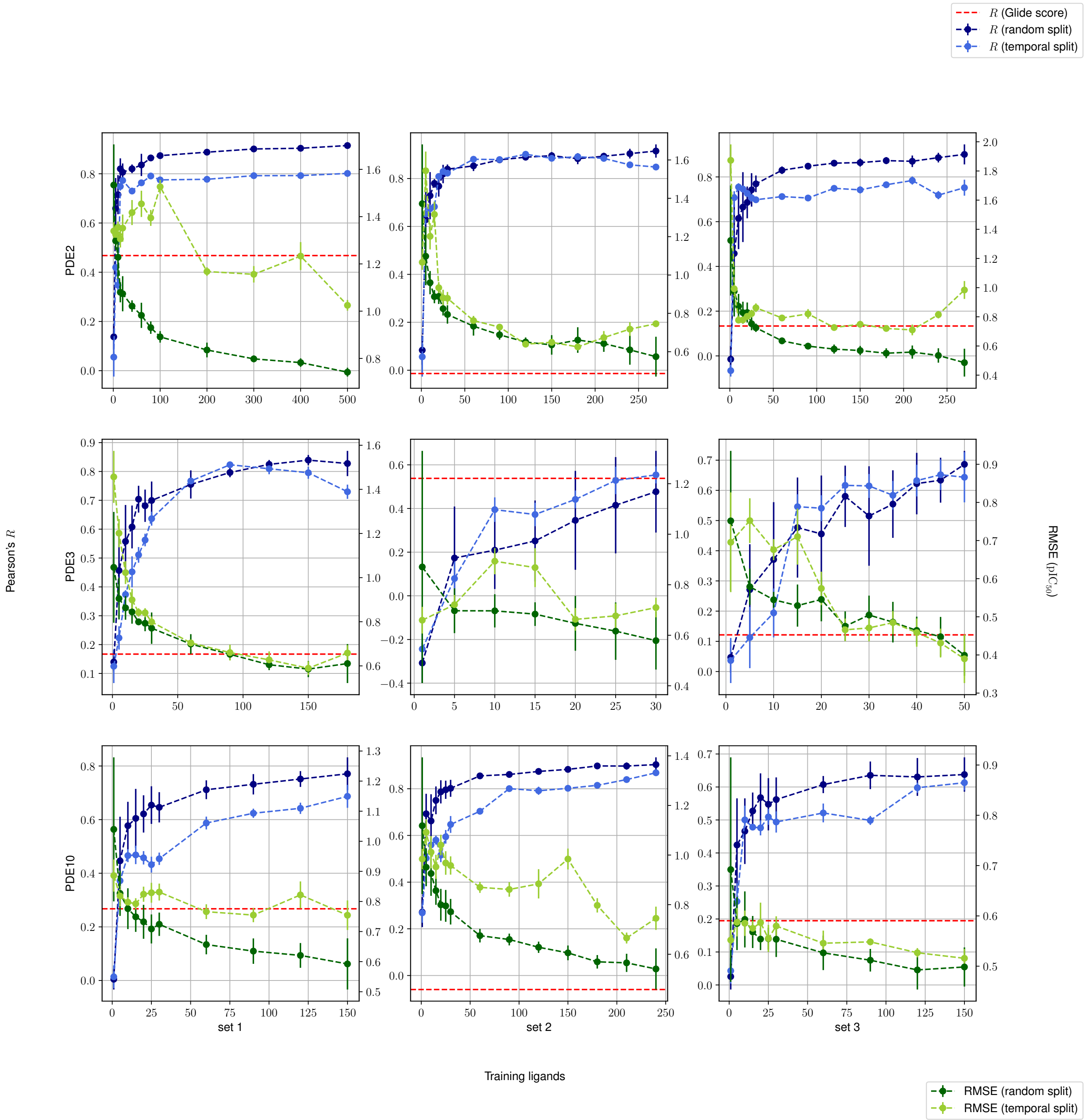

Supplementary Figure 3: Average Pearson's correlation coefficient R and RMSE (± 1 standard deviation) based on 25 independent runs on the Janssen PDE sets, using both a random and a temporal split and a Glide score baseline.

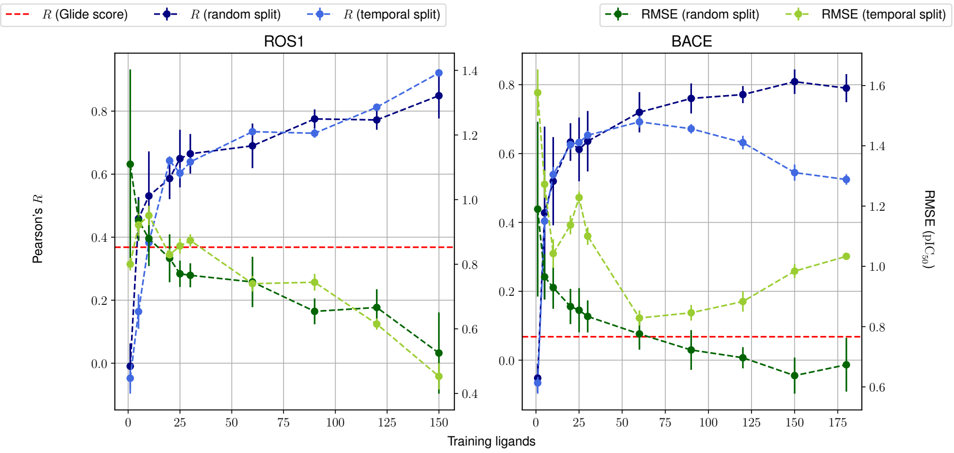

Supplementary Figure 4: Average Pearson's correlation coefficient R and RMSE (± 1 standard deviation) based on 25 independent runs on the Janssen ROS1 and BACE sets, using both a random and a temporal split and a Glide score baseline.

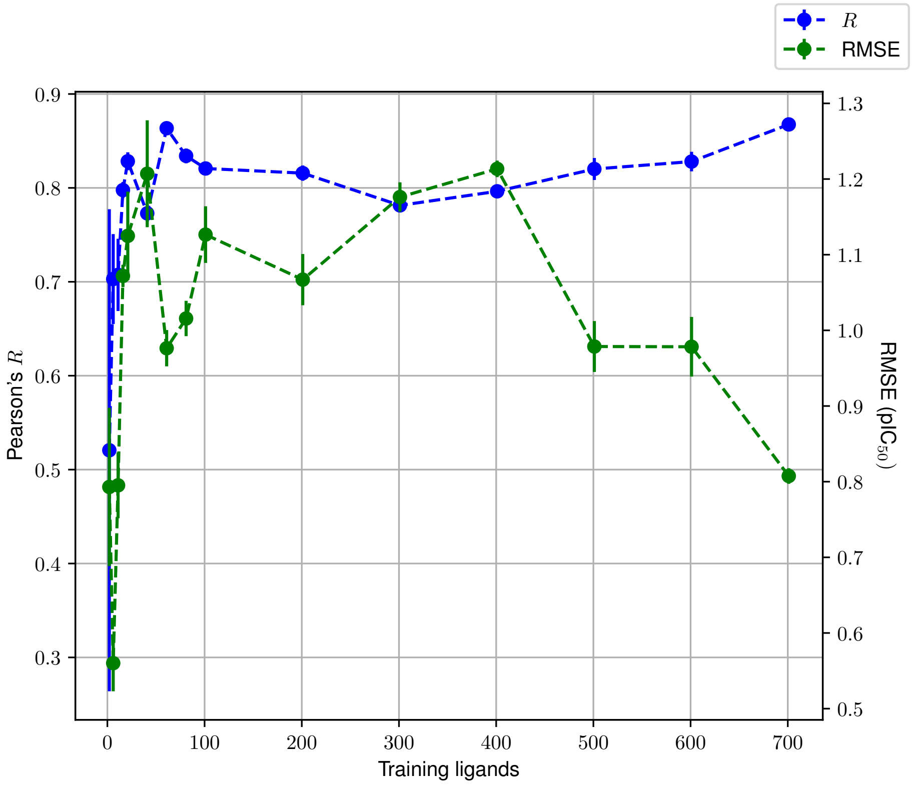

Supplementary Figure 5: Pearson's correlation coefficient R and RMSE (± 1 standard deviation) based on 25 independent runs on the Janssen PDE2 first set using a chemical similarity based split.

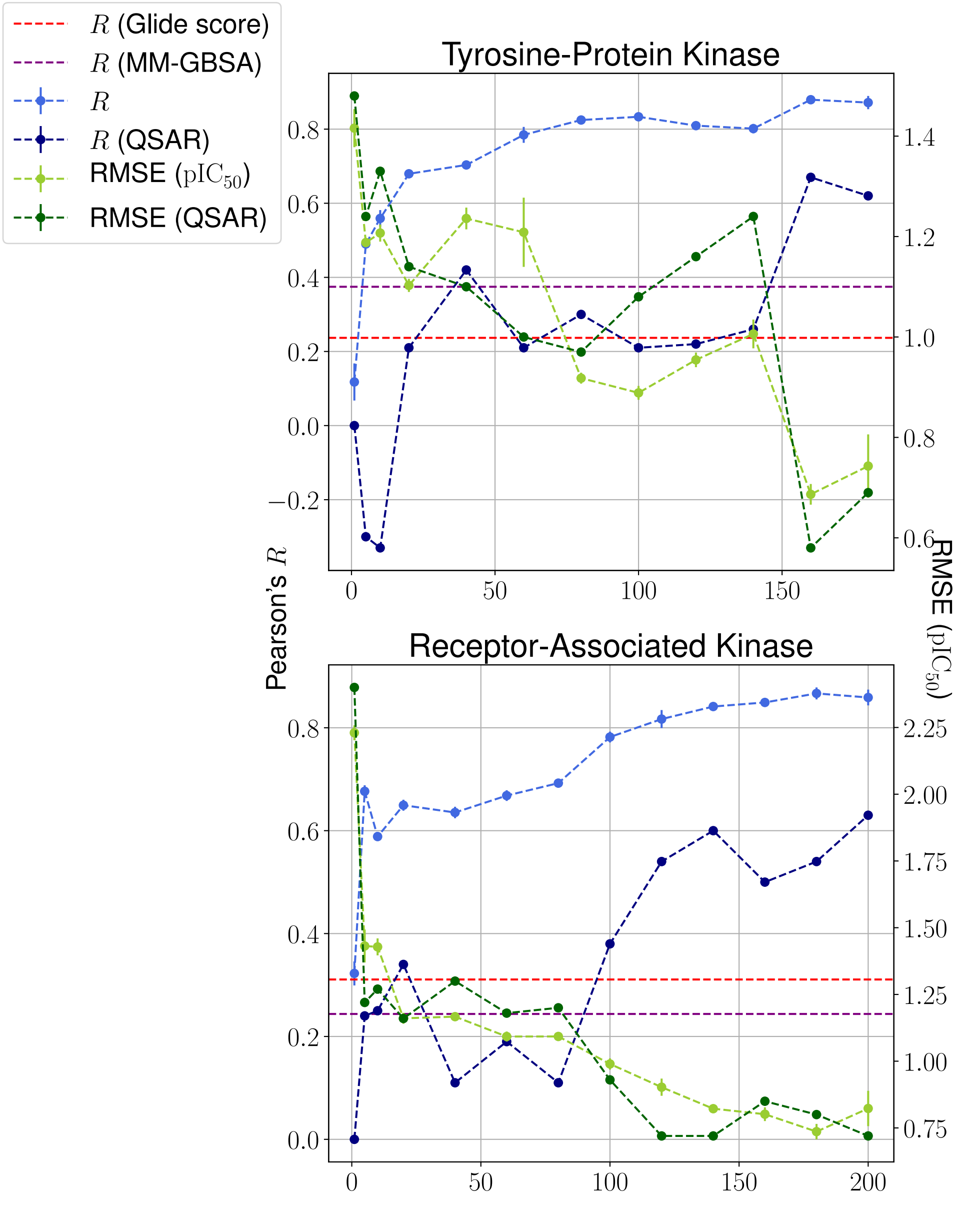

Supplementary Figure 6: Average Pearson's correlation coefficient R and RMSE (± 1 standard deviation) based on 5 independent runs on the Biogen Tyrosine-Protein Kinase and Receptor-Associated Kinase sets, using temporal split and with MM-GBSA, Glide score, and a random forest QSAR model (MACCS + ECFP4 + rdkit descriptors) as baselines.

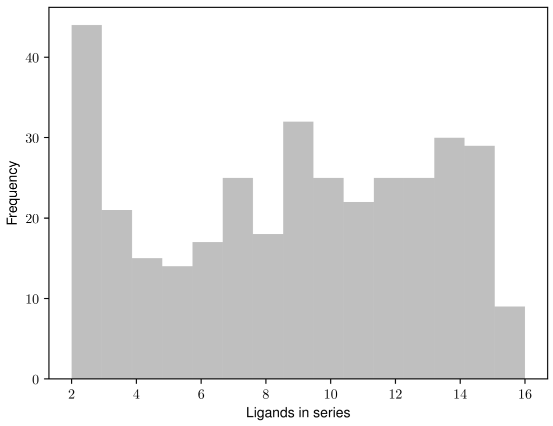

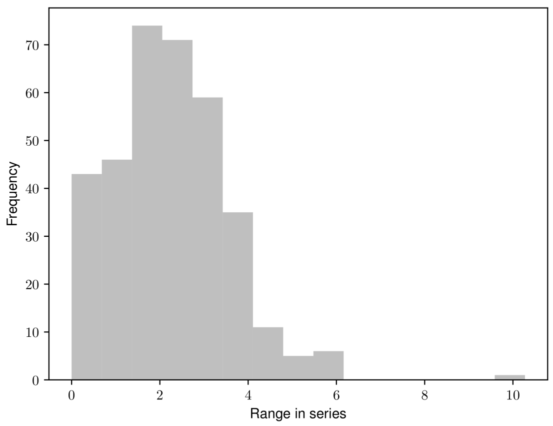

Supplementary Figure 7: Histograms of available ligands and range per congeneric series in the BindingDB database.

References

[1] Lingle Wang, Yujie Wu, Yuqing Deng, Byungchan Kim, Levi Pierce, Goran Krilov, Dmitry Lupyan, Shaughnessy Robinson, Markus K. Dahlgren, Jeremy Greenwood, Donna L. Romero, Craig Masse, Jennifer L. Knight, Thomas Steinbrecher, Thijs Beuming, Wolfgang Damm, Ed Harder, Woody Sherman, Mark Brewer, Ron Wester, Mark Murcko, Leah Frye, Ramy Farid, Teng Lin, David L. Mobley, William L. Jorgensen, Bruce J. Berne, Richard A. Friesner, and Robert Abel. Accurate and Reliable Prediction of Relative Ligand Binding Potency in Prospective Drug Discovery by Way of a Modern Free-Energy Calculation Protocol and Force Field. *Journal of the American Chemical Society*, 137(7):2695–2703, 2015.

[2] David L. Mobley and Michael K. Gilson. Predicting Binding Free Energies: Frontiers and Benchmarks. *Annual Review of Biophysics*, 46(1):531–558, 2017.

[3] Germano Heinzelmann, Niel M. Henriksen, and Michael K. Gilson. Attach-Pull-Release Calculations of Ligand Binding and Conformational Changes on the First BRD4 Bromodomain. *Journal of Chemical Theory and Computation*, 13(7):3260–3275, 2017.

[4] Matteo Aldeghi, Alexander Heifetz, Michael J. Bodkin, Stefan Knapp, and Philip C. Biggin. Accurate Calculation of the Absolute Free Energy of Binding for Drug Molecules. *Chemical Science*, 7(1):207–218, 2016.
